# Vitamin and Carotenoid Intake and Outcomes of In Vitro Fertilization in Women Referring to an Italian Fertility Service: A Cross-Sectional Analysis of a Prospective Cohort Study

**DOI:** 10.3390/antiox12020286

**Published:** 2023-01-27

**Authors:** Valentina De Cosmi, Sonia Cipriani, Giovanna Esposito, Francesco Fedele, Irene La Vecchia, Giuseppe Trojano, Fabio Parazzini, Edgardo Somigliana, Carlo Agostoni

**Affiliations:** 1 Department of Clinical Sciences and Community Health, Università degli Studi di Milano, 20122 Milan, Italy; 2 Department of Obstetrics and Gynecology, Fondazione IRCCS Ca’ Granda Ospedale Maggiore Policlinico, 20122 Milano, Italy; 3 Infertility Unit, Fondazione IRCCS Ca’ Granda Ospedale Maggiore Policlinico, 20122 Milano, Italy; 4 Department of Maternal and Child Health, “Madonna delle Grazie” Hospital ASM, 75100 Matera, Italy; 5 SC Pediatria-Immunoreumatologia, Fondazione IRCCS Ca’ Granda Ospedale Maggiore Policlinico, 20122 Milan, Italy

**Keywords:** nutrition, antioxidants, assisted reproductive techniques, infertility

## Abstract

Background: Nutrition may impact reproductive health and fertility potential. The role of dietary antioxidants in affecting conception and birth outcomes is a topic of emerging interest. Methods: This cross-sectional analysis from a prospective cohort study aims to explore the relationship between the intake of antioxidants, vitamins, and carotenoids and the outcomes of assisted reproduction techniques. Information on the socio-demographic characteristics, health histories, lifestyle habits, and diet information of subfertile couples referred to a fertility center was obtained. Results: A total of 494 women were enrolled. According to the four IVF outcomes considered, 95% of women achieved good quality oocytes, 87% achieved embryo transfer, 32.0% achieved clinical pregnancies, and 24.5% achieved pregnancy at term. Associations were found between age and the number of good quality oocytes (*p* = 0.02). A moderate level of physical activity in the prior 5 years was associated with a better rate of achieving clinical pregnancy (*p* = 0.03). Smoking habits, alcohol intake, and caffeine consumption did not show associations with any outcome. No associations were found, even after accounting for potential confounders, with the intake of vitamins C, D, E, and α-carotene, β-carotene, beta-cryptoxanthin, lutein, and folate. Conclusion: Further research is needed to understand how antioxidant intake may have a role in modulating fertility.

## 1. Introduction

Nutritional factors may contribute in significant ways to determining reproductive health and thus influencing fertility in humans [1]. The role of nutrients in processes such as implantation, placental growth, and angiogenesis is well-recognized, as is the transfer of nutrients from the mother to the fetus. Up to 15% of couples experience infertility worldwide, particularly in developed countries [2]. Unbalanced nutrition is defined as excessive energy intake as well as inadequate micronutrient and macronutrient intake. Malnutrition, together with the reduction in physical activity, contributes to body fat increase and to the development of non-communicable diseases [3]. Lifestyle factors, such as smoking, alcohol abuse, and poor nutritional intake, can also negatively impact fertility potential [4]. 

The role of dietary antioxidant intake in affecting conception and birth outcome is a topic of emerging interest [5]. Oxidative stress (OS) and low antioxidant status may be associated with infertility of both known and idiopathic origins [5]. Growing evidence indicates that OS impairs gamete and embryo development outcomes during assisted reproductive procedures techniques (ART) [6]. A lower total antioxidant status has been reported in infertile women [7]. Thus, it has been suggested that supplementation with antioxidants may improve infertility treatment outcomes [8]. 

Vitamin C, α- and β-carotene, vitamin E, and β-cryptoxanthin have antioxidant activities and may thus provide cellular defense against reactive oxygen species (ROS) that damage DNA [9]. Folate is important for the synthesis of DNA, transfer RNA, and the amino acids cysteine and methionine, and folate is a relevant contributor of reproduction. Folic acid, in the form of folate, effectively scavenges oxidizing free radicals, and, as such, it too can be regarded as an antioxidant [10]. 

Various randomized controlled trials (RCTs) have investigated the role of antioxidant supplementation and of a good antioxidant’s status, but findings are still inconsistent. For instance, Ruder et al. showed that vitamin C, β-carotene, and vitamin E were related to better ART outcomes in subgroups of women defined by BMI or age [11]. At the same time, another group of authors found that vitamin C supplementation increased pregnancy rates primarily among non-smokers [12]. Notably, the high intake of some vitamins (e.g., vitamin A) may result in adverse reproductive outcomes. This has led to the hypothesis that relation between micronutrients and ART outcomes may not be linear [13]. In vitro and animal studies have focused on several possible ways through which OS may affect fertility and early pregnancy loss. However, no study has directly addressed the effects of OS on fertility in women, although a recent review has addressed the effects of folate, zinc, and antioxidants in the pathogenesis of subfertility [14]. Studying diet in relation to fertility outcome raises interest, as it is a potentially modifiable predictor of a treatment outcome. The present study elaborates on the relationship between lifestyle and micronutrient intake with four main outcomes: (1) good quality oocytes, (2) successful embryo transfer, (3) clinical pregnancies, and (4) successful term pregnancies in women of subfertile couples presenting to an Italian Fertility Clinic and candidates for in vitro fertilization (IVF). 

## 2. Materials and Methods

### Sample Size

The number of 494 patients allows a power (1-β) of 0.77, 0.89, 0.86, and 0.66, for incidences of the reference group of 0.2, 0.4, 0.6, and 0.8, respectively, assuming a relative risk = 1.5 and α = 0.05. The calculations are the same as those in the comparing proportions for two independent samples setting: p1 = p0 and p2 = p0 * RR/(1 + p0 * (RR − 1)) [15].

Methods for this study are described elsewhere [16]. Briefly, both partners of couples who agreed to participate were interviewed by trained personnel. Information on general socio-demographic characteristics, personal and health histories, and habits (including smoking, physical activity, alcohol intake, and methylxanthine-containing beverage consumption) was collected. 

Through a reproducible and validated food frequency questionnaire (FFQ), information on diet was obtained. The FFQ included 78 foods items or various food groups—such as the major sources of animal fats (i.e., red meat, milk, cheese, ham, and salami), folate, vitamins (vegetables and fruit), pasta and bread, cake, sweets, chocolate, and fish—and the most common Italian recipes [9,10,11]. Patients were asked to report their usual weekly food consumption in the previous year. The FFQ included the average weekly consumption of 78 food items or food groups. Energy levels and mineral intake, as well as macronutrient and micronutrient intake, were estimated using the most recent update of an Italian food consumption database [12]. 

Subjects were interviewed on their usual consumption of alcoholic beverages. Considering the different ethanol concentrations, one drink corresponded to approximately 125 mL of wine, 330 mL of beer, and 30 mL of hard liquor (i.e., about 12.5 g of ethanol). Total alcohol intake, expressed in grams of ethanol per day (g/day), was calculated as the sum of all reported alcoholic beverages. Patients who abstained from drinking for life were categorized as “never drinkers”; individuals who had abstained from drinking for at least 12 months at the time of the interview were categorized as “ex-drinkers.” For this study, we considered the subjects in this category as “abstainers.”

Information on coffee and other methylxanthine-containing beverages (tea, cocoa, and decaffeinated coffee), was also collected, and the average number of cups consumed per day was registered. Caffeine intake from coffee (60 mg per cup), cappuccino (75 mg per cup), tea (45 mg per cup), decaffeinated coffee (4 mg per cup), and chocolate (6 mg/10 g) was calculated [13]. Satisfactory reproducibility of questions on self-reported smoking and drinking habits in our study populations was previously reported [14]. Patients were managed according to a standardized clinical protocol [17]. A serum hCG assessment to detect pregnancy was performed 14 or 16 days after an ovulation triggering or luteinizing hormone (LH) surge. Women with positive human chorionic gonadotropin (hCG) values underwent a transvaginal sonography three weeks later. Clinical pregnancy was defined as the presence of at least one intrauterine gestational sac. 

We report data on the female partners in relation to the first IVF outcome. The study was proposed during the diagnostic phase, and the interview was administered on the oocyte retrieval day to collect information on general socio-demographic characteristics, health histories, and lifestyle habits. Couples that did not speak Italian were excluded. 

The participation rate was close to 95%. Body mass index (BMI) was classified through the World Health Organization (WHO) indications [18]. Occupational physical activity (PA) was categorized as heavy/very heavy, light/moderate, and mainly standing or mainly sitting. Leisure PA was defined in terms of hours/week: <2, between 2 and 4, and ≥5. We noted the smoking habits collected as never, former, or current; we also noted the number of cigarettes smoked daily and the duration of smoking. Information on alcohol and caffeine intake was also collected, as previously reported. A previously validated FFQ [19,20,21] was used to obtain information on diet. Using information obtained from a validated database, we estimated the macronutrient, calorie, and micronutrient intakes, particularly of vitamins (C, D, E), α-carotene, β-carotene, beta-cryptoxanthin, lutein, and folate. The content of each micronutrient in the foods was calculated as follows: frequency of consumption of the food, multiplied by the portion of the food consumed given from the FFQ questionnaire, multiplied by the mean content of the micronutrient in the specific food. The FFQ explored the average weekly consumption of 78 food items or food groups and beverages per year. Intakes that were lower than once per week, but at least as high as once per month, were coded as 0.5 per week. To account for seasonal consumption, we considered weekly consumption of vegetables/fruits available in limited periods during the year and weighted for months of consumption. Daily energy and mineral, macronutrient, and micronutrient intakes were estimated using the most recent update of an Italian food composition database [22].

## 3. Results

In total, 494 women were included in the present study. The lifestyles, anthropometric characteristics, and clinical characteristics of the women are presented in Table 1 according to the considered IVF outcomes. 

The mean (SD) age was 36.9 (3.6) years (range 27–46 years). The mean (SD) BMI was 22.4 (4.0) kg/m^2^ (range 17.0–42.0 kg/m^2^). Twenty-nine (5.9%) women were obese (BMI > 30 kg/m^2^). Considering the four IVF outcomes, 469 women (95%) achieved good quality oocytes, 429 women (87%) achieved embryo transfer, 158 women (32.0%) achieved clinical pregnancies, and 121 women (24.5%) achieved pregnancy at term. Associations were found between age and the number of good quality oocytes (*p* = 0.0223). Women aged 35–39 years had a higher number of good quality oocytes and a higher rate of achieving clinical pregnancy and pregnancy at term (*p* = 0.0223 and *p* < 0.0001, respectively). Moderate physical activity in the prior 5 years (2–4 h of sport per week) was associated with a better rate of achieving clinical pregnancy (*p* = 0.0359). Among other lifestyle indicators, smoking habits, alcohol intake, and caffeine consumption did not show associations with any outcome. Descriptive statistics are reported in Table 2. 

Adjusted relative risks (ARRs) and 95% CI for vitamins (C, D, E), α-carotene, β-carotene, beta-cryptoxanthin, lutein and folate are reported in Table 3. Considering all four outcomes, and each antioxidant, no associations were found, including after accounting for potential confounders. Higher concentrations of the follicle-stimulating hormone were not associated with the number of good quality oocytes (*p* = 0.1111). Follicle-stimulating hormone concentrations were negatively associated with embryo transfer, clinical pregnancy, and pregnancy at term (*p* < 0.0001).

The Anti-Müllerian hormone (AMH) concentrations and the number of oocytes used for retrieval were associated with all the IVF outcomes considered (all *p* < 0.0001) (Table 4).

## 4. Discussion

In a cohort of Italian women, we examined whether the intake of vitamins C, D, E, and α-carotene, β-carotene, beta-cryptoxanthin, lutein, and folate is associated with four outcomes of ART. Our results do not find any associations between these micronutrients and any of the IVF outcomes considered. We find evidence of association between a moderate level of physical activity in the prior 5 years and a better rate of achieving clinical pregnancy. No associations emerge with the other lifestyle indicators. It is well demonstrated how lifestyle factors affect fertility, and inappropriate diet or insufficient dietary micronutrients may manifest itself in subfertility. Similarly to our results on physical activity, Chavarro et al. showed that a combination of adequate diet, physical activity, and weight control was associated with a 69% lower risk of subfertility due to ovulatory disorders [23].

Micronutrients play independent roles for the development of the fetus and the prevention of pregnancy-related complications [24]. There is biological plausibility to hypothesize that these micronutrients, sharing anti-inflammatory activities, may be helpful in infertility treatment. The intensive metabolism of granulosa cells and the high numbers of macrophages and neutrophilic granulocytes in the follicle wall at ovulation may point to active generation of Reactive Oxygen Species (ROS). ROS levels, within certain physiological ranges, may be necessary for the normal development of the oocyte and embryo growth; however, high levels may indicate OS. Animal studies have demonstrated that deficiencies of vitamins A, C, and D result in diminished fertility [25,26]. Studies of men indicate that diet is crucial in preventing oxidative damage to sperm DNA [27]. Furthermore, antioxidants have been shown to be associated with reproductive hormone concentrations in healthy women, suggesting potentially complex hormonal interactions [28].

In results similar to ours, Ming-Chieh Li et al. [13] followed 349 women undergoing ART cycles for infertility treatment. The authors found that the total consumption of vitamins A, C, and E prior to starting infertility treatment with ART was not associated with live birth rates. Other results raised unexpected inverse associations of β-carotene intake from foods and of lutein with live birth rates. Conversely, Ruder et al. reported that a higher intake of β-carotene was related to a longer time to pregnancy among women over 35 years who participated in an RCT of infertility treatment, and the opposite relation was observed among younger women [11]. Moreover, a prospective cohort study of 1228 women measured preconception levels of, among other variables, cryptoxanthin, α- and β-carotene, and α- and γ-tocopherol in serum. The authors found that higher preconception serum carotenoid concentrations were associated with improved fecundability and a shorter time to pregnancy among women with no history of infertility. By contrast, serum γ-tocopherol levels that were at or above the US average were associated with a longer time to pregnancy. A positive association between carotenoids and shortened time to pregnancy was also reported in a recent study of women diagnosed with unexplained infertility [11]. Regarding the folate, supplemental folate, at intake levels that are much higher than those currently recommended, was related to a higher probability of live birth among women undergoing ART [29]. 

Most of the literature in this field concerns the investigation of the effect of supplementation with mixtures of antioxidants or other supplements that include antioxidants; however, epidemiologic studies and RCTs focusing on single antioxidants, including vitamins A, C, and E, or carotenoids, are limited and produced inconsistent findings. A 2017 Cochrane review of RCTs concluded that among women undergoing ART, antioxidants were not associated with an increased live birth rate, and this review did not draw strong conclusions regarding the effects of supplementation of antioxidants on ART outcomes [8].

The heterogeneity of the FFQ used, as well as the inter-variability of food composition tables, which may even differ year to year, may at least partly explain these differing findings [30].

Clear dietary recommendations have not yet been established for preconception health, except for the assumption of 400 mcg/day of folic acid, to prevent defects of the neural tube development. Specific supplements, except for folic acid, are not routinely advocated by gynecologists before IVF. Consequently, during the study period, supplements were not routinely prescribed in our clinical center. Furthermore, the interest of the lay press in the role of supplements in improving fertility has only increased more recently; thus, it is conceivable that only a very limited proportion of women considered in this study were using supplements. Some surveys have shown that healthier dietary patterns, characterized by better adherence to the Mediterranean diet—with high consumption of legumes, whole grains, vegetables, and fruits (therefore rich in vitamins C, D, E, as well as α-carotene, β-carotene, beta-cryptoxanthin, lutein, and folate)—and lower intakes of both processed foods and sugar-sweetened beverages, are associated with shorter time to pregnancy, higher fecundability, higher rates of clinical pregnancy and live birth, and a reduced risk of ovulatory disorder infertility [31,32,33].

A strength of our report is represented by its prospective design and the high participation rate in our relatively large sample size in a monocentric survey. However, some limitations should be noted. First, our clinical center, at the time of the study, did not advise the use of antioxidants or other supplements except for folic acid, and we did not collect any information on the type of supplement that the women were using prior to the IVF treatment. Consequently, all of our results on the consumption of micronutrients are related to the micronutrients derived from food sources. Second, measurement bias was inevitable, and it may have occurred because participants may underestimate or overestimate their dietary intakes. Finally, these results may not be generalizable to women without known fertility problems.

## 5. Conclusions

Subfertile women are highly motivated to explore all paths of treatment in their desire to have a healthy baby. Antioxidants are mostly unregulated and are readily available for purchase by consumers. Women should therefore be made more aware of the role of lifestyle changes and an overall healthy diet. Reliable knowledge about the role of nutrition on fertility is far from complete, and more long-term investigations with larger cohorts are recommended to confirm our findings. However, up to the availability of robust interventional evidence, our results argue against the use of antioxidants in women requiring IVF.

## Figures and Tables

**Table 1:** Baseline characteristics by IVF outcomes.

	Good Quality Oocytes					Embryo Transfer					Clinical Pregnancy					Pregnancy at Term *				
	No N = 25		Yes N = 469			No N = 65		Yes N = 429			No N = 336		Yes N = 158			No N = 372		Yes N = 121		
	N	%	N	%	*p*-Value **	N	%	N	%	*p*-Value **	N	%	N	%	*p*-Value **	N	%	N	%	*p*-Value **
Age (years)	3	12.0	133	28.4	0.0223	10	15.4	126	29.4	0.0540	79	23.5	57	36.1	0.0004	87	23.4	49	40.5	<0.0001
1. <35																				
2. 35–39	11	44.0	234	49.9		36	55.4	209	48.7		165	49.1	80	50.6		186	50.0	58	47.9	
3. ≥40	11	44.0	102	21.7		19	29.2	94	21.9		92	27.4	21	13.3		99	26.6	14	11.6	
Education	11	44.0	228	48.6	0.6528	28	43.1	211	49.2	0.3585	161	47.9	78	49.4	0.7635	181	48.7	57	47.1	0.7672
1. College																				
2. Degree	14	56.0	241	51.4		37	56.9	218	50.8		175	52.1	80	50.6		191	51.3	64	52.9	
BMI					0.6933 ***					0.3883					0.9705					0.5923
<18.5	1	4.0	41	8.7		5	7.7	37	8.6		29	8.6	13	8.2		35	9.4	7	5.8	
18.5–24.9	17	68.0	335	71.4		47	72.3	305	71.1		241	71.7	111	70.3		261	70.2	91	75.2	
25.0–29.9	5	20.0	63	13.4		11	16.9	57	13.3		45	13.4	23	14.6		52	14.0	15	12.4	
30.0+	1	4.0	28	6.0		1	1.5	28	6.5		19	5.7	10	6.3		22	5.9	7	5.8	
Missing	1	4.0	2	0.4		1	1.5	2	0.5		2	0.6	1	0.6		2	0.5	1	0.8	
Smoking habit	15	60.0	258	55.0	0.7627	34	52.3	239	55.7	0.8722	188	56.0	85	53.8	0.6049	210	56.5	63	52.1	0.6024
Never																				
Current	5	20.0	86	18.3		13	20.0	78	18.2		64	19.0	27	17.1		68	18.3	22	18.2	
Former	5	20.0	125	26.7		18	27.7	112	26.1		84	25.0	46	29.1		94	25.3	36	29.8	
Physical activity (5 years—work)					0.9531					0.2753					0.9397					0.9643
1. (Very) heavy/moderate	7	28.0	139	29.6		22	33.8	124	28.9		100	29.8	46	29.1		111	29.8	34	28.1	
3. Mainly standing	5	20.0	100	21.3		9	13.8	96	22.4		73	21.7	32	20.3		79	21.2	26	21.5	
4. Mainly sitting	13	52.0	228	48.6		34	52.3	207	48.3		163	48.5	78	49.4		182	48.9	59	48.8	
Missing	0	-	2	0.4		0	-	2	0.5		0	-	2	1.3		0	-	2	1.7	
Physical activity (5 years—sport)					0.6433					0.9701					0.0359					0.0550
1. > 5 h/week	2	8.0	69	14.7		10	15.4	61	14.2		57	17.0	14	8.9		61	16.4	10	8.3	
3. 2–4 h/week	11	44.0	190	40.5		26	40.0	175	40.8		128	38.1	73	46.2		144	38.7	57	47.1	
4. <2 h/week	12	48.0	208	44.3		29	44.6	191	44.5		150	44.6	70	44.3		166	44.6	53	43.8	
Missing	0	-	2	0.4		0	-	2	0.5		1	0.3	1	0.6		1	0.3	1	0.8	
Physical activity (1 year—work)					0.6425					0.4306					0.8070					0.8103
1. (Very) heavy/moderate	5	20.0	134	28.6		20	30.8	119	27.7		93	27.7	46	29.1		102	27.4	36	29.8	
3. Mainly standing	6	24.0	100	21.3		10	15.4	96	22.4		75	22.3	31	19.6		82	22.0	24	19.8	
4. Mainly sitting	14	56.0	233	49.7		35	53.8	212	49.4		168	50.0	79	50.0		188	50.5	59	48.8	
Missing	0	-	2	0.4		0	-	2	0.5		0	-	2	1.3		0	-	2	1.7	
Physical activity (1 year—sport)					0.6632					0.3091					0.0244					0.1535
1. > 5 h/week	2	8.0	49	10.4		10	15.4	41	9.6		43	12.8	8	5.1		44	11.8	7	5.8	
3. 2–4 h/week	11	44.0	165	35.2		20	30.8	156	36.4		113	33.6	63	39.9		128	34.4	47	38.8	
4. <2 h/week	12	48.0	254	54.2		35	53.8	231	53.8		180	53.6	86	54.4		200	53.8	66	54.5	
Missing	.	.	1	0.2		.	.	1	0.2		.	.	1	0.6		.	.	1	0.8	
Alcohol	6	24.0	134	28.6	0.9598	16	24.6	124	28.9	0.6220	92	27.4	48	30.4	0.1163	102	27.4	38	31.4	0.3755
1. no alcohol intake																				
2. 0.1–2.36 g/day	6	24.0	112	23.9		14	21.5	104	24.2		72	21.4	46	29.1		84	22.6	33	27.3	
3. 2.36–5.85 g/day	7	28.0	115	24.5		16	24.6	106	24.7		91	27.1	31	19.6		98	26.3	24	19.8	
4. >5.85 g/day	6	24.0	108	23.0		19	29.2	95	22.1		81	24.1	33	20.9		88	23.7	26	21.5	
Caffeine intake	10	40.0	153	32.6	0.3647	21	32.3	142	33.1	0.8563	107	31.8	56	35.4	0.6692	119	32.0	44	36.4	0.2781
1. 0–81.9 g/week																				
2. 82.0–179.0 g/week	10	40.0	158	33.7		24	36.9	144	33.6		118	35.1	50	31.6		134	36.0	34	28.1	
3. ≥180.0 g/week;	5	20.0	158	33.7		20	30.8	143	33.3		111	33.0	52	32.9		119	32.0	43	35.5	
Calories (kcal/day)	9	36.0	155	33.0	0.9542	20	30.8	144	33.6	0.7854	108	32.1	56	35.4	0.1478	120	32.3	43	35.5	0.3812
1. 0–1525.7																				
2. 1525.8–1923.9	8	32.0	158	33.7		21	32.3	145	33.8		107	31.8	59	37.3		122	32.8	44	36.4	
3. 1924–3344	8	32.0	156	33.3		24	36.9	140	32.6		121	36.0	43	27.2		130	34.9	34	28.1	
FSH (mIU/mL)					0.1111					<0.0001					0.0001					<0.0001
<5.80	5	20.0	114	24.3		11	16.9	108	25.2		71	21.1	48	30.4		81	21.8	38	31.4	
5.80–7.29	3	12.0	123	26.2		9	13.8	117	27.3		79	23.5	47	29.7		87	23.4	39	32.2	
7.30–8.89	5	20.0	117	24.9		12	18.5	110	25.6		79	23.5	43	27.2		89	23.9	32	26.4	
≥8.9	11	44.0	115	24.5		32	49.2	94	21.9		106	31.5	20	12.7		114	30.6	12	9.9	
Missing	1	4.0	0	-		1	1.5	0	-		1	0.3	0	-		1	0.3	0	-	
AMH (ng/mL)					0.0010					0.0011					<0.0001					<0.0001
<0.81-	12	48.0	109	23.2		26	40.0	95	22.1		100	29.8	21	13.3		108	29.0	13	10.7	
0.81–1.59	8	32.0	107	22.8		17	26.2	98	22.8		81	24.1	34	21.5		88	23.7	26	21.5	
1.60–3.19	3	12.0	123	26.2		10	15.4	116	27.0		85	25.3	41	25.9		92	24.7	34	28.1	
≥3.20	0	-	124	26.4		8	12.3	116	27.0		65	19.3	59	37.3		77	20.7	47	38.8	
Missing	2	8.0	6	1.3		4	6.2	4	0.9		5	1.5	3	1.9		7	1.9	1	0.8	
Oocytes used (number)					<0.0001					<0.0001					<0.0001					<0.0001
<3.0	25	100.0	87	18.6		49	75.4	63	14.7		103	30.7	9	5.7		106	28.5	6	5.0	
3.00–4.99	0	-	121	25.8		3	4.6	118	27.5		89	26.5	32	20.3		100	26.9	21	17.4	
5.00–7.99	0	-	127	27.1		9	13.8	118	27.5		76	22.6	51	32.3		88	23.7	38	31.4	
≥8.00	0	-	134	28.6		4	6.2	130	30.3		68	20.2	66	41.8		78	21.0	56	46.3	

Abbreviations: AMH, anti-Müllerian hormone; BMI, body mass index; FSH, follicle-stimulating hormone; NE, Not evaluable; * one missing ** *p*-value(Chi-square Test).

**Table 2:** Antioxidant and micronutrient intake of 494 women according to IVF outcomes.

Variable	Good Quality Oocytes				Embryo Transfer				Clinical Pregnancy				Pregnancy at Term			
	No (25)		Yes (469)		No (65)		Yes (429)		No (336)		Yes (158)		No (372)		Yes (121)	
	Median	Q1–Q3	Median	Q1–Q3	Median	Q1–Q3	Median	Q1–Q3	Median	Q1–Q3	Median	Q1–Q3	Median	Q1–Q3	Median	Q1–Q3
Vitamin C mg/day	125.4	91–167	127.7	94–167	123.6	90–168	127.8	94–167	130.0	94–172	122.7	94–157	128.4	94–171	123.8	92–153
Vitamin D µg/day	3.2	3–4	3.2	2–4	3.2	2–4	3.2	2–4	3.2	2–4	3.2	2–4	3.2	2–4	3.1	2–4
Vitamin E mg/day	11.2	10–13	10.8	9–14	11.4	10–14	10.7	9–14	10.9	9–14	10.6	8–14	10.9	9–14	10.6	8–14
α-carotene µg/day	763.8	537–1246	742.5	453–1344	778.4	526–1295	732.4	447–1344	761.4	485–1370	685.3	410–1272	758.6	469–1342	683.9	410–1363
β-carotene µg/day	4356.8	3309–5484	4170.3	3156–5842	4224.4	3298–6099	4170.3	3156–5819	4389.9	3189–5868	3966.7	3156–5763	4348.6	3221–5839	3869.0	3031–5886
β-cryptoxanthin µg/day	144.6	121–288	194.9	108–291	145.2	92–277	207.4	109–292	192.2	109–293	198.1	106–288	194.1	110–293	194.1	90–287
Lutein µg/day	355.8	269–429	308.8	235–420	314.0	236–429	310.2	235–420	308.1	236–415	314.3	224–425	308.2	234–415	315.6	240–425
Folate µg/day	210.7	195–239	217.9	184–259	211.7	190–258	220.3	185–258	223.2	187–262	209.0	181–249	221.1	188–261	208.3	176–248

**Table 3:** Adjusted relative risks (ARR) and 95% confidence intervals (CI) for percentiles of selected food intake.

	Good Quality Oocytes					Embryo Transfer					Clinical Pregnancy					Pregnancy at Term				
Variable ^§^	Yes	%	ARR *	95% CI		Yes	%	ARR **	95% CI		Yes	%	ARR ***	95% CI		Yes	%	ARR ***	95% CI	
Vitamin C mg/day																				
1st (29.8–94.1)	116	94.3	1+			104	84.6	1+			39	31.7	1+			32	26.0	1+		
2nd (94.1–127.6)	118	95.2	1.01	0.95	1.07	109	87.9	1.04	0.94	1.15	46	37.1	1.17	0.83	1.65	31	25.2	0.97	0.63	1.48
3rd (127.6–167.4)	118	95.9	1.02	0.96	1.08	110	89.4	1.06	0.96	1.17	42	34.1	1.08	0.75	1.54	36	29.3	1.12	0.75	1.69
4th (167.4–471.2)	117	94.4	1.00	0.94	1.06	106	85.5	1.01	0.91	1.12	31	25.0	0.79	0.53	1.18	22	17.7	0.68	0.42	1.10
Vitamin D µg/day																				
1st (0.3–2.3)	118	95.9	1+			106	86.2	1+			35	28.5	1+			28	22.8	1+		
2nd (2.3–3.2)	117	94.4	0.98	0.93	1.04	109	87.9	1.02	0.93	1.12	43	34.7	1.22	0.84	1.76	33	26.6	1.17	0.75	1.81
3rd (3.2–4.0)	118	95.9	1.00	0.95	1.05	109	88.6	1.03	0.94	1.13	36	29.3	1.03	0.69	1.52	27	22.1	0.97	0.61	1.55
4th (4.0–9.5)	116	93.5	0.98	0.92	1.03	105	84.7	0.98	0.89	1.09	44	35.5	1.25	0.86	1.80	33	26.6	1.17	0.75	1.81
Vitamin E mg/day																				
1st (3.9–8.6)	119	96.7	1+			108	87.8	1+			45	36.6	1+			36	29.5	1+		
2nd (8.6–10.9)	119	96.0	0.99	0.94	1.04	113	91.1	1.04	0.95	1.13	36	29.0	0.79	0.55	1.14	26	21.0	0.71	0.46	1.10
3rd (10.9–13.7)	112	91.1	0.94	0.88	1.00	100	81.3	0.93	0.83	1.03	37	30.1	0.82	0.58	1.17	28	22.8	0.77	0.50	1.18
4th (13.7–30.0)	119	96.0	0.99	0.94	1.04	108	87.1	0.99	0.90	1.09	40	32.3	0.88	0.62	1.25	31	25.0	0.85	0.56	1.28
α-carotene µg/day																				
1st (17.9–458.6)	118	95.9	1+			109	88.6	1+			44	35.8	1+			33	26.8	1+		
2nd(458.6–742.6)	117	94.4	0.98	0.93	1.04	109	87.9	0.99	0.91	1.09	41	33.1	0.92	0.65	1.30	32	26.0	0.97	0.64	1.47
3rd (742.6–1344.2)	117	94.4	0.98	0.93	1.04	104	83.9	0.95	0.86	1.05	37	29.8	0.83	0.58	1.19	25	20.2	0.75	0.48	1.19
4th (1344.2–5604.7)	117	95.1	0.99	0.94	1.05	107	87.0	0.98	0.89	1.08	36	29.3	0.82	0.57	1.18	31	25.2	0.94	0.62	1.43
β-carotene µg/day																				
1st (613.2–3168.8)	118	95.9	1+			109	88.6	1+			41	33.3	1+			34	27.6	1+		
2nd (3168.8–4194.6)	117	94.4	0.98	0.93	1.04	106	85.5	0.96	0.88	1.06	47	37.9	1.14	0.81	1.59	34	27.6	1.00	0.67	1.50
3rd (4194.6–5841.5)	116	94.3	0.98	0.93	1.04	108	87.8	0.99	0.90	1.09	31	25.2	0.76	0.51	1.12	22	17.9	0.65	0.40	1.04
4th (5841.5–17908.9)	118	95.2	0.99	0.94	1.05	106	85.5	0.96	0.88	1.06	39	31.5	0.94	0.66	1.35	31	25.0	0.90	0.60	1.37
β-cryptoxanthin µg/day																				
1st (1.0–108.0)	117	95.1	1+			105	85.4	1+			41	33.3	1+			33	26.8	1+		
2nd (108.0–194.1)	116	93.5	0.98	0.93	1.05	99	79.8	0.94	0.83	1.05	36	29.0	0.87	0.60	1.26	28	22.6	0.84	0.54	1.30
3rd (194.1–290.9)	118	95.9	1.01	0.96	1.06	113	91.9	1.08	0.98	1.18	48	39.0	1.17	0.84	1.63	39	32.0	1.19	0.81	1.76
4th (290.9–1240.2)	118	95.2	1.00	0.95	1.06	112	90.3	1.06	0.96	1.16	33	26.6	0.80	0.54	1.17	21	16.9	0.63	0.39	1.03
Lutein µg/day																				
1st (72.0–234.8)	118	95.2	1+			108	87.1	1+			41	33.1	1+			29	23.6	1+		
2nd (234.8–311.1)	119	96.7	1.02	0.97	1.07	108	87.8	1.01	0.92	1.11	33	26.8	0.81	0.55	1.19	27	22.0	0.93	0.59	1.48
3rd (311.1–420.3)	116	93.5	0.98	0.92	1.04	107	86.3	0.99	0.90	1.09	43	34.7	1.05	0.74	1.49	33	26.6	1.13	0.73	1.74
4th (420.3–945.0)	116	94.3	0.99	0.93	1.05	106	86.2	0.99	0.90	1.09	41	33.3	1.01	0.71	1.44	32	26.0	1.10	0.71	1.71
Folate µg/day																				
1st (76.5–185.4)	118	95.9	1+			107	87.0	1+			44	35.8	1+			36	29.5	1+		
2nd (185.4–217.6)	113	91.1	0.95	0.89	1.01	101	81.5	0.94	0.84	1.04	46	37.1	1.04	0.75	1.44	32	25.8	0.87	0.58	1.31
3rd (217.6–258.1)	119	96.7	1.01	0.96	1.06	113	91.9	1.06	0.97	1.15	36	29.3	0.82	0.57	1.18	28	22.8	0.77	0.50	1.18
4th (258.1–434.3)	119	96.0	1.00	0.95	1.05	108	87.1	1.00	0.91	1.10	32	25.8	0.72	0.49	1.06	25	20.2	0.68	0.44	1.07

^§^ The cut-off points of the quartiles of daily intake for the micronutrients are expressed in brackets.First reference category for all variables. * Adjusted for age. ** Adjusted for age and BMI. *** Adjusted for age and prior 5 years of leisure physical activity.

**Table 4:** Concentrations of Anti-Müllerian hormone (AMH) and follicle-stimulating hormone (FSH) and number of oocytes used for retrieval according to IVF outcomes.

Variable	Good Quality Oocytes					Embryo Transfer					Clinical Pregnancy					Pregnancy at Term				
	No (25)		Yes (469)			No (65)		Yes (429)			No (336)		Yes (158)			No (372)		Yes (121)		
	Median	Q1–Q3	Median	Q1–Q3	*p* *	Median	Q1-Q3	Median	Q1-Q3	*p* *	Median	Q1–Q3	Median	Q1-Q3	*p* *	Median	Q1–Q3	Median	Q1–Q3	*p* *
FSH (mIU/mL)	8.6	6.4–11.5	7.2	5.8–8.8	0.0241	8.9	6.8–11.1	7.0	5.7–8.6	<0.0001	7.8	6.1–9.8	6.7	5.4–8.1	<0.0001	7.6	6.0–9.6	6.5	5.3–7.9	<0.0001
AMH (ng/mL)	0.8	0.3–1.4	1.7	0.9–3.3	<0.0001	1.0	0.4–1.9	1.7	0.9–3.4	<0.0001	1.4	0.7–2.8	2.2	1.2–3.9	<0.0001	1.5	0.7–2.8	2.3	1.2–3.9	<0.0001
Number of oocytes used	0.0	0.0–0.0	5.0	3.0–8.0	<0.0001	1.0	0.0–2.0	5.0	3.0–8.0	<0.0001	4.0	2.0–7.0	7.0	4.0–9.0	<0.0001	4.0	2.0–7.0	7.0	5.0–10.0	<0.0001

* 1-sided non-parametric Wilcoxon test.

## Data Availability

The raw data supporting the conclusions article will be made available by the authors without undue reservation.
